# Supplementary figures and images for: Combination of bortezomib and venetoclax targets the pro-survival function of LMP-1 and EBNA-3C of Epstein-Barr virus in spontaneous lymphoblastoid cell lines
Source: PLoS Pathog. 2024 Sep 26;20(9):e1012250. doi: 10.1371/journal.ppat.1012250 (PMC11481030; doi:10.1371/journal.ppat.1012250)

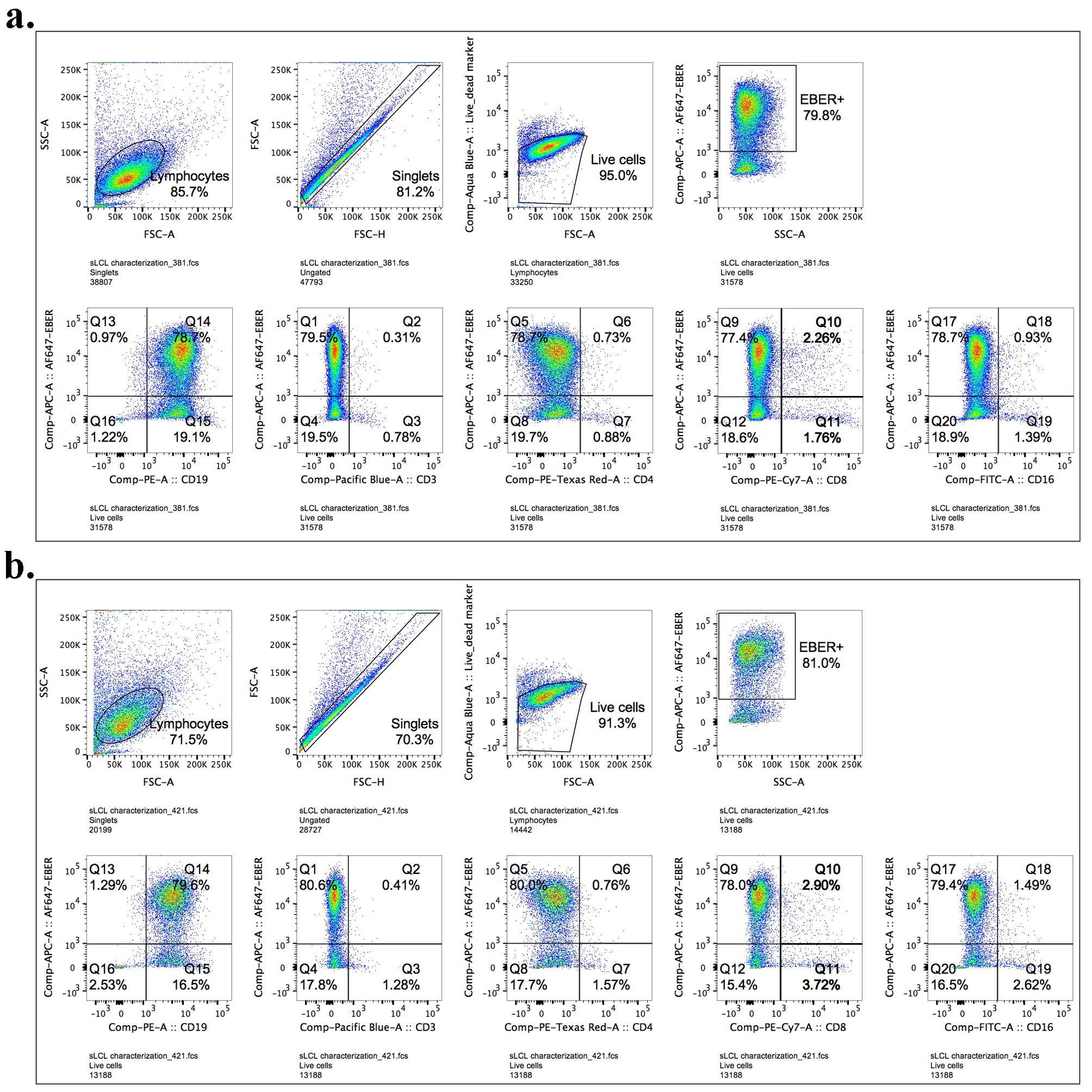

Supplement: S1 Fig — 1.5 x 10^6^ (a) sLCL 381 and (b) sLCL 421 cells were stained by Aqua Blue dye (Invitrogen, USA) and the following fluorochrome-conjugated antibodies: Pacific Blue anti-human CD3, PE-Cy7 anti-human CD8, PE anti-human CD19, FITC anti-human CD16/56 (BioLegend, USA) and PE-Texas Red anti-CD4 (eBioscience, USA) on ice for 30 minutes for indication of cell viability and immunophenotype. The cells were fixed by fixation buffer and subsequently treated with PrimeFlow RNA permeabilization buffer with RNase inhibitors. The EBER in the cells were stained by fluorochrome-conjugated probe EBER-AF647 (Thermo Fisher Scientific, USA) and the cells were incubated in the dark at 4°C overnight. On the next day, the cells were incubated with PrimeFlow RNA PreAmp Mix, PrimeFlow RNA Amp Mix, and diluted Label Probes for signal amplification. The samples were measured by flow cytometry and analyzed by FlowJo software (Tree Star). (TIF) [file ppat.1012250.s001.tif]

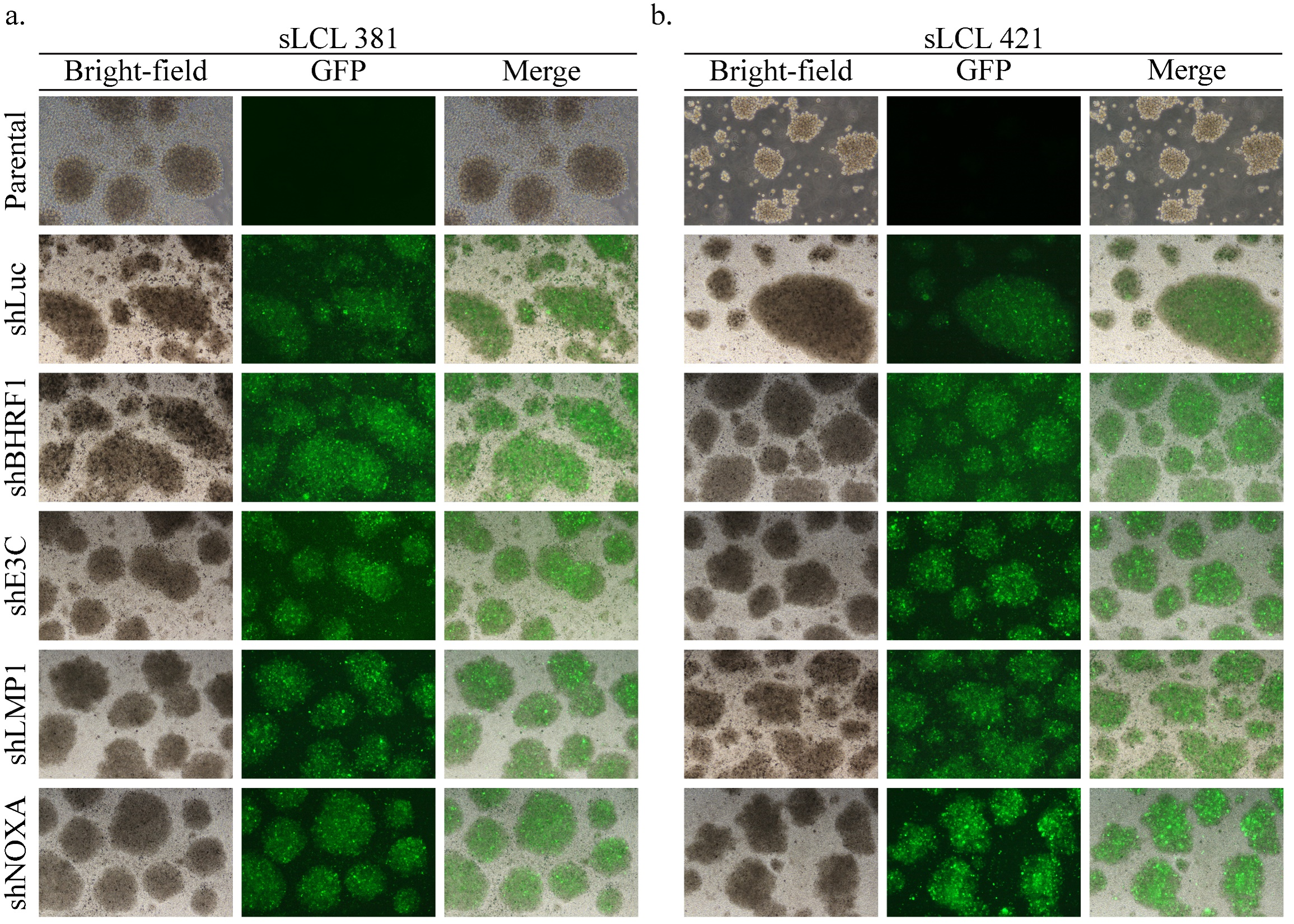

Supplement: S2 Fig — (a) sLCL 381 panel and (b) sLCL 421 panel transduced with lentivirus of either shLuc (scrambled control), shBHRF1, shE3C, shLMP1 or shNOXA and under puromycin selection (2 μg/ml) for at least 8 weeks were illustrated. Parental cells mean the sLCLs without transduction of lentivirus. (TIF) [file ppat.1012250.s002.tif]

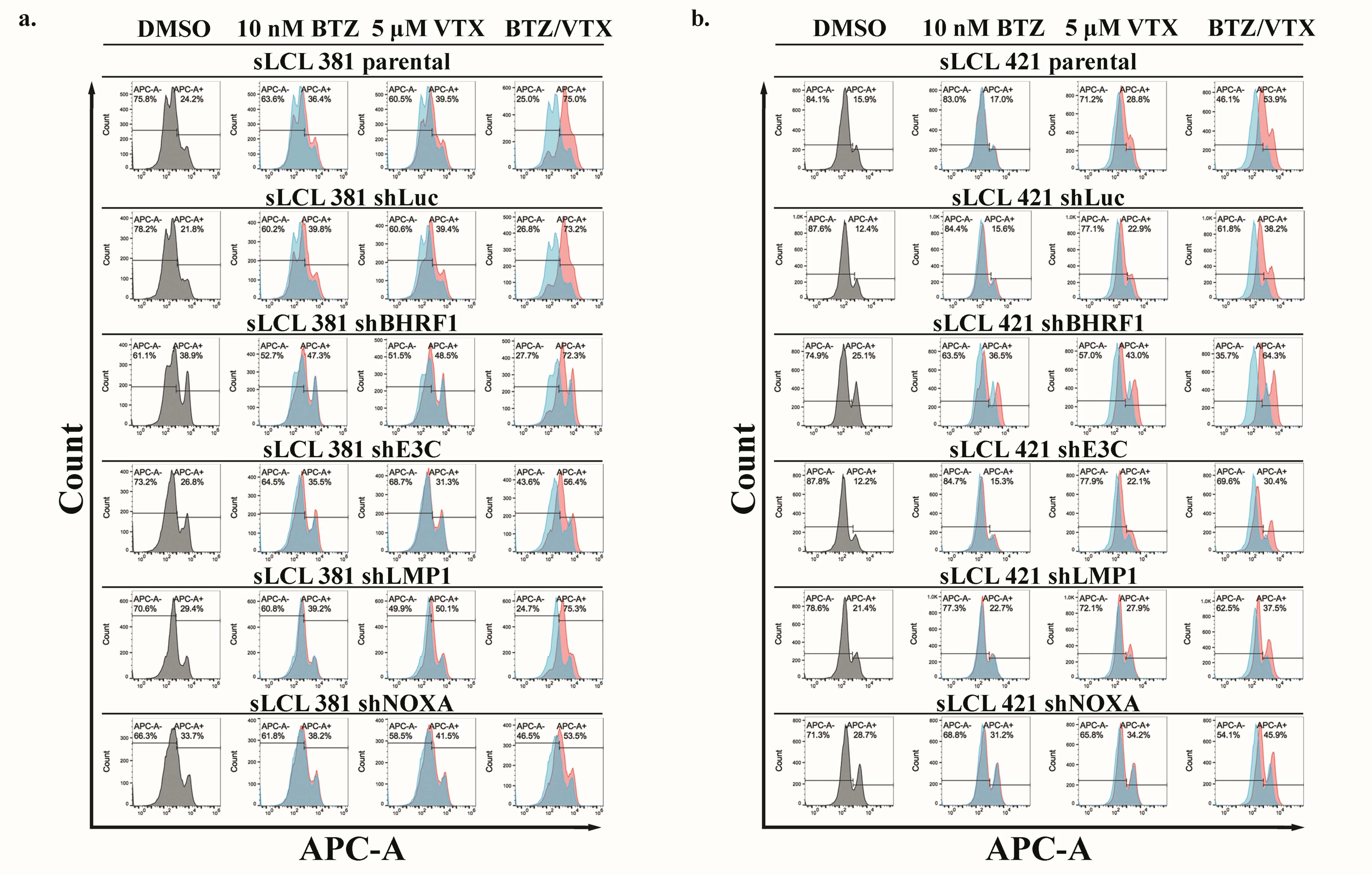

Supplement: S3 Fig — (a) sLCL 381 and (b) sLCL 421 panels were either treated with DMSO, 10 nM bortezomib, 5 μM venetoclax or bortezomib/venetoclax for 24 hours. The cells were harvested and fixed by 10% neutral buffered formalin (NBF) and 70% ethanol at -20°C overnight. The DNA break was labelled by TdT and BrdU, which was detected by APC-conjugated anti-BrdU antibody and measured by flow cytometry. One representative set of TUNEL assays of the cell lines upon treatments was presented. (TIF) [file ppat.1012250.s003.tif]

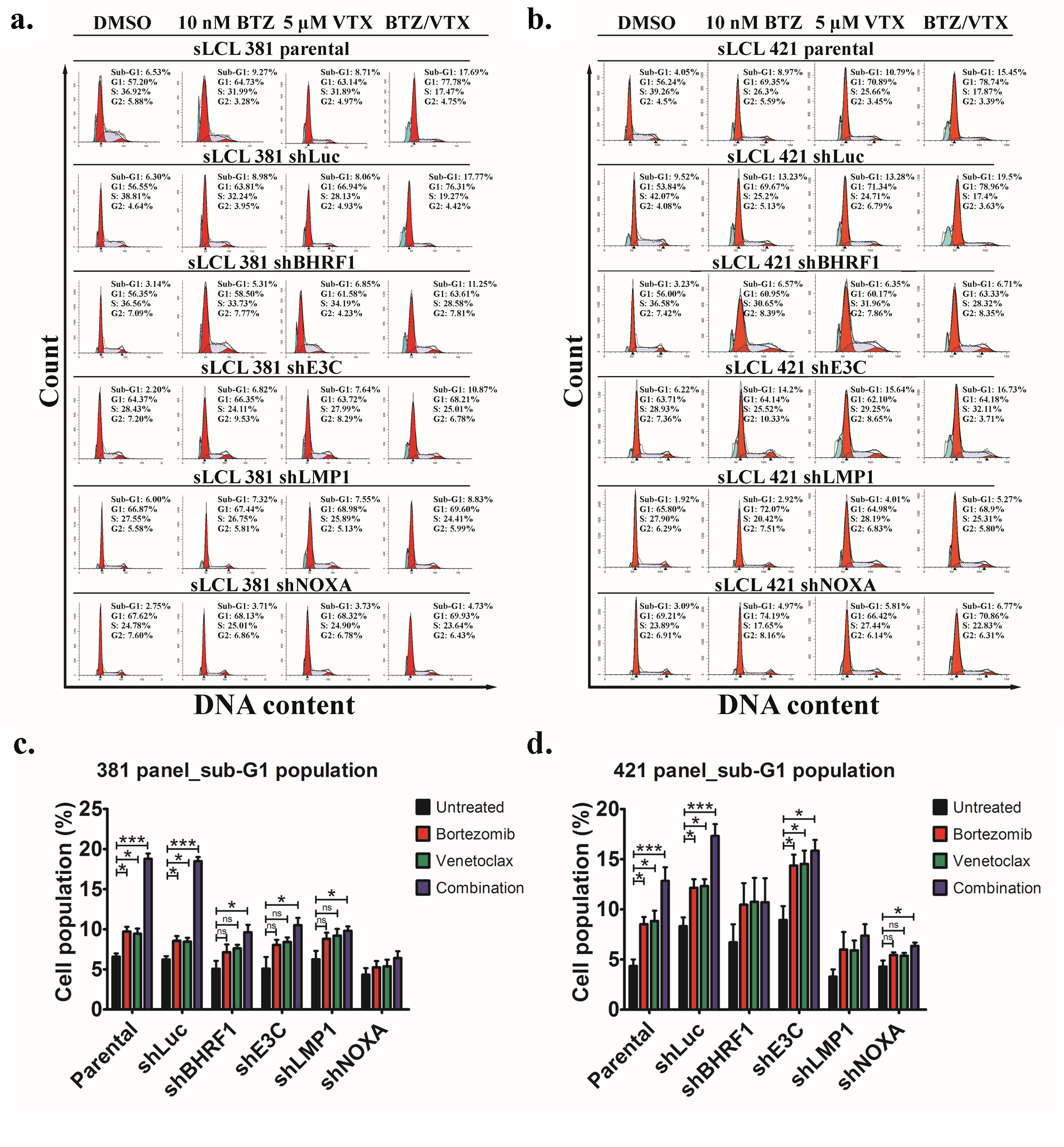

Supplement: S4 Fig — (a) sLCL 381 and (b) sLCL 421 panels were either treated with DMSO, 10 nM bortezomib, 5 μM venetoclax or bortezomib/venetoclax for 24 hours. The next day, the cells were incubated with 500 μg/ml RNase for 10 minutes, followed by propidium iodide (PI) staining. The cellular DNA content was measured by flow cytometry and analyzed by ModFit LT 3.0. One representative set of cell cycle patterns of the cell lines upon treatments was presented. (c) Percentages of sLCL 381 and (d) 421 cells in sub-G1 phases. The results were analyzed for statistical significance using One-way ANOVA Dunnett’s Multiple Comparison Test. p-value less than 0.05 was considered statistically significant; *p < 0.05, **p < 0.01, ***p < 0.001, ns = not significant. Error bars represent the standard error of mean (SEM) of data obtained from three independent experiments. (TIF) [file ppat.1012250.s004.tif]

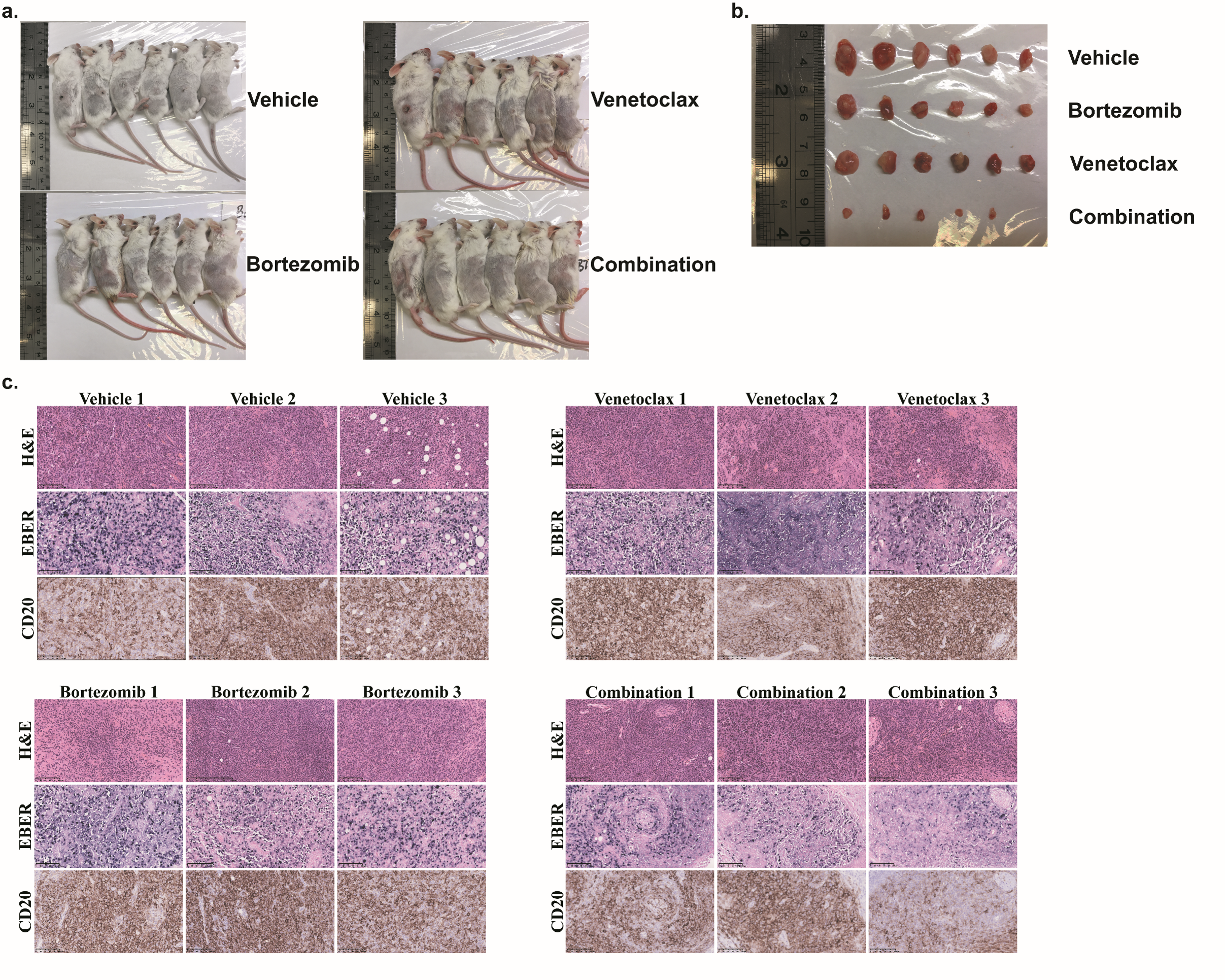

Supplement: S5 Fig — sLCL 381 (1 x 10^7^ cells) were subcutaneously injected into the right flanks of SCID mice, at age of 6–7 weeks. When the mean volume of the tumors reached to approximate 50 mm3, the mice were treated with either 1 mg/kg bortezomib (intraperitoneal (IP) injection in 2% DMSO, 30% PEG (polyethylene glycol) 400 (MedChemExpress, USA) and 69% saline on day 1, 5 and 9, n = 6), 100 mg/kg venetoclax (oral gavage (PO) in 5% DMSO, 60% phosal 50PG (MedChemExpress, USA), 30% PEG400 and 5% ethanol for 5 days per week until day 22, n = 6), bortezomib/venetoclax (n = 6), or DMSO vehicle control (n = 6). (a) The mice were euthanized by 150 mg/kg 0.6% pentobarbital via IP injection and (b) the tumors were dissected out at the end of experiment (30 days post-treatment). (c) Histological analysis confirmed that the tumour cells expressed B-cell marker CD20 by immunohistochemistry, and EBV-encoded small RNA (EBER) by in-situ hybridization (scale bar: 100 μm with 20 μm intervals). (TIF) [file ppat.1012250.s005.tif]

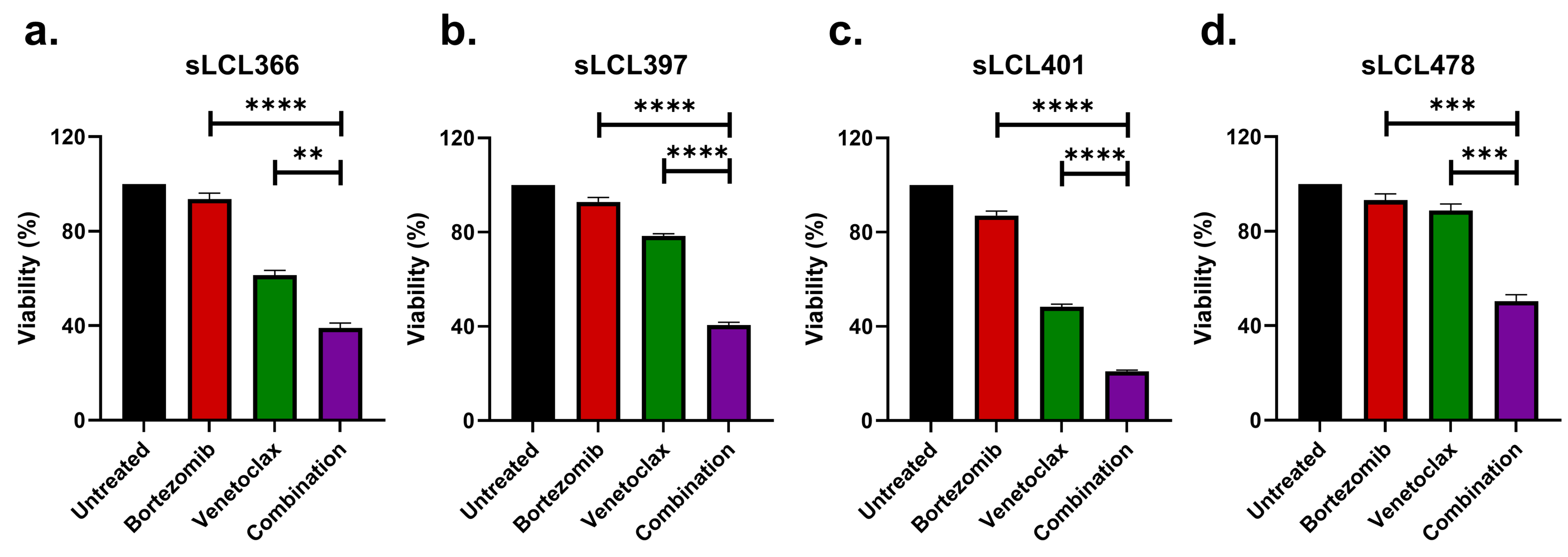

Supplement: S6 Fig — (a) sLCL 366, (b) sLCL 397, (c) sLCL 401, and (d) sLCL 478 were treated with 10nM of bortezomib and 5μM of venetoclax for 24 hours and stained by CCK8 kit. OD450nm was measured, and cell viability was plotted. **p < 0.01, ***p < 0.001, ****p < 0.0001. (TIF) [file ppat.1012250.s006.tif]

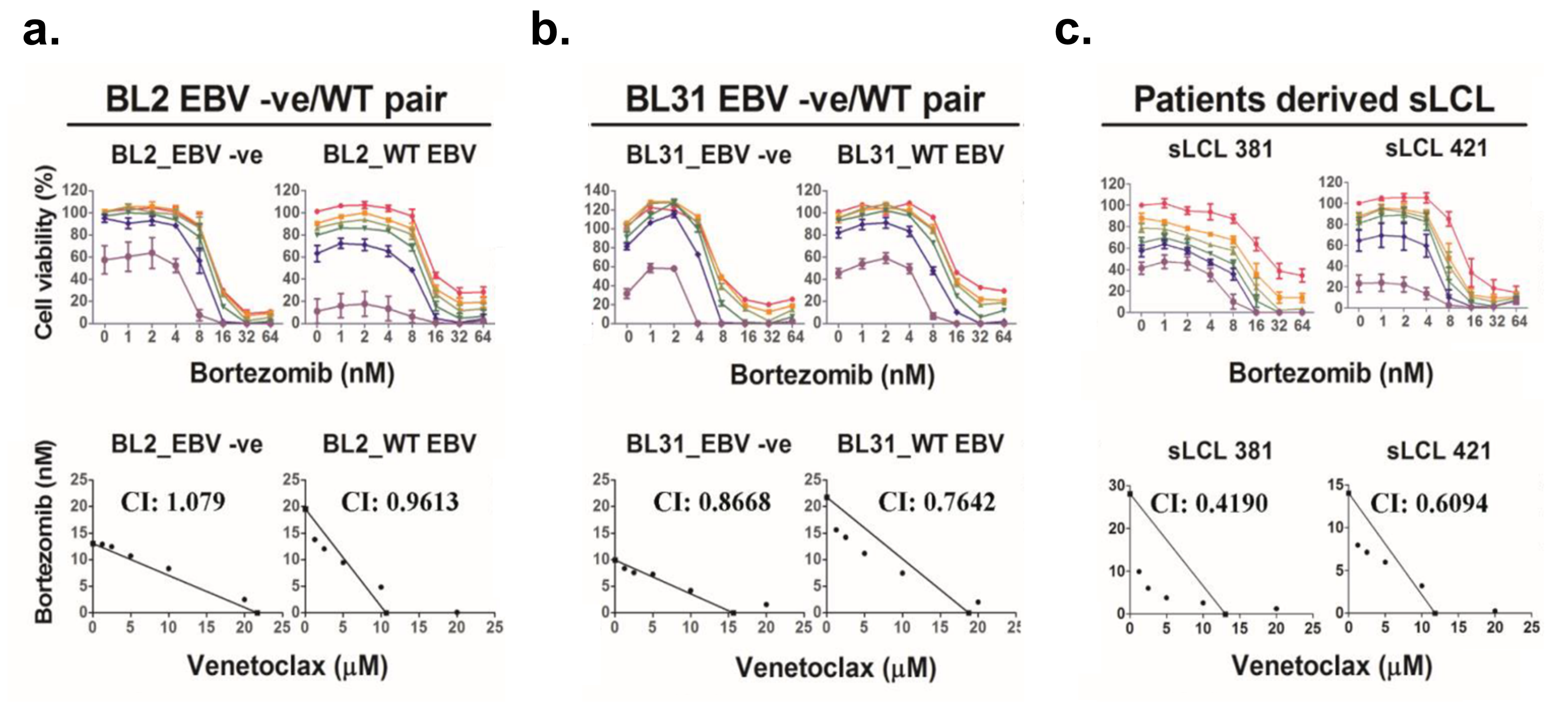

Supplement: S7 Fig — The cells were treated with combination of bortezomib (0, 1, 2, 4, 8, 16, 32, and 64 nM) and venetoclax (0, 1.25, 2.5, 5, 10, 20 μM) for 24 hours and stained by MTT solution. OD570 nm and OD630 nm were measured, and cell viability was plotted. Percentages of cell viability of (a) BL2 EBV-negative/WT EBV pair, (b) BL31 EBV-negative/WT EBV pair, and (c) sLCL381 and sLCL 421 in bort/venetoclax treatments were determined, and isobolograms were applied for analysis of synergism. (TIF) [file ppat.1012250.s007.tif]

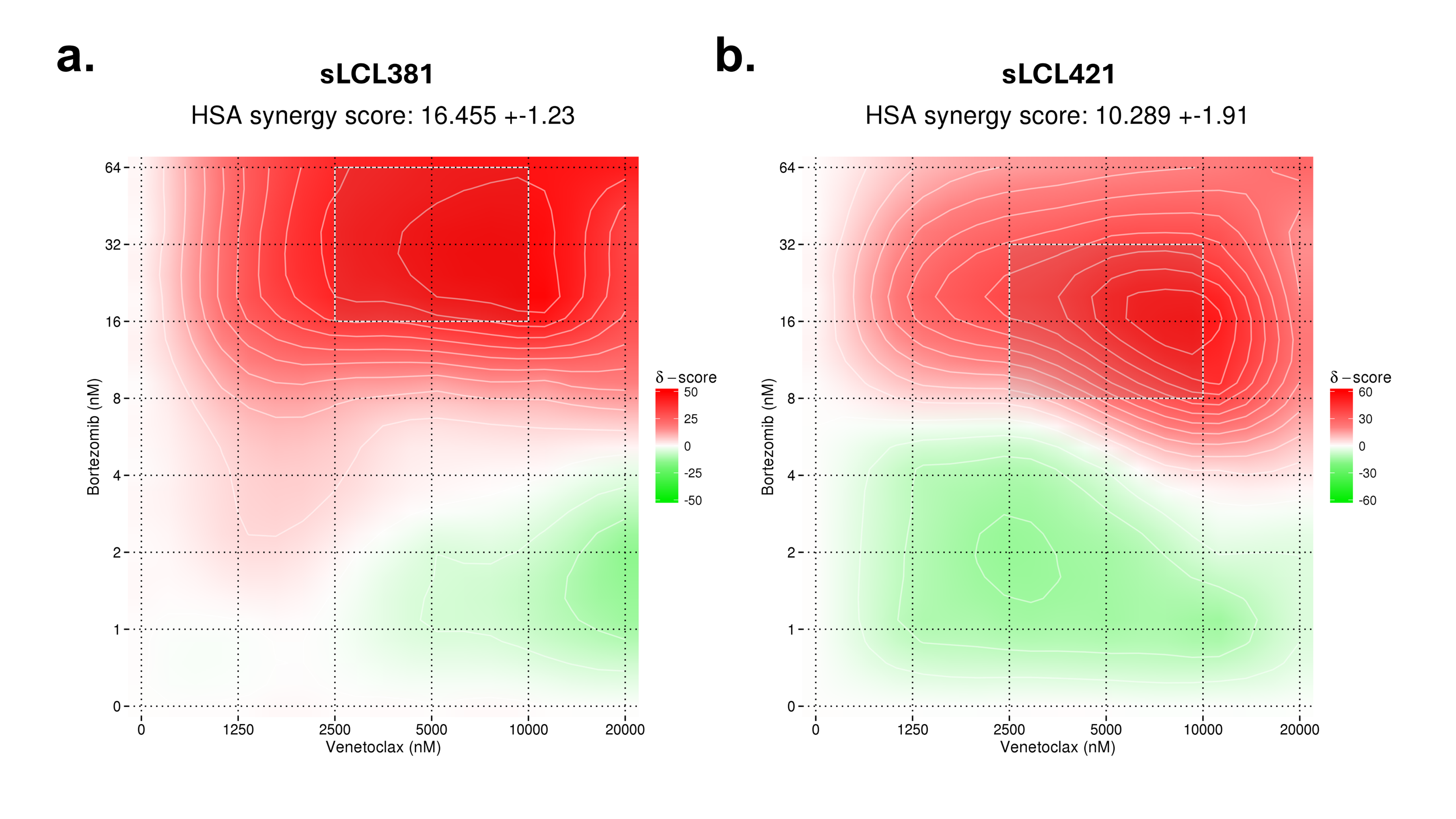

Supplement: S8 Fig — The viability of (a) sLCL 381 and (b) sLCL 421 were loaded to SynergyFinder and the synergy scores were computed. (TIF) [file ppat.1012250.s008.tif]

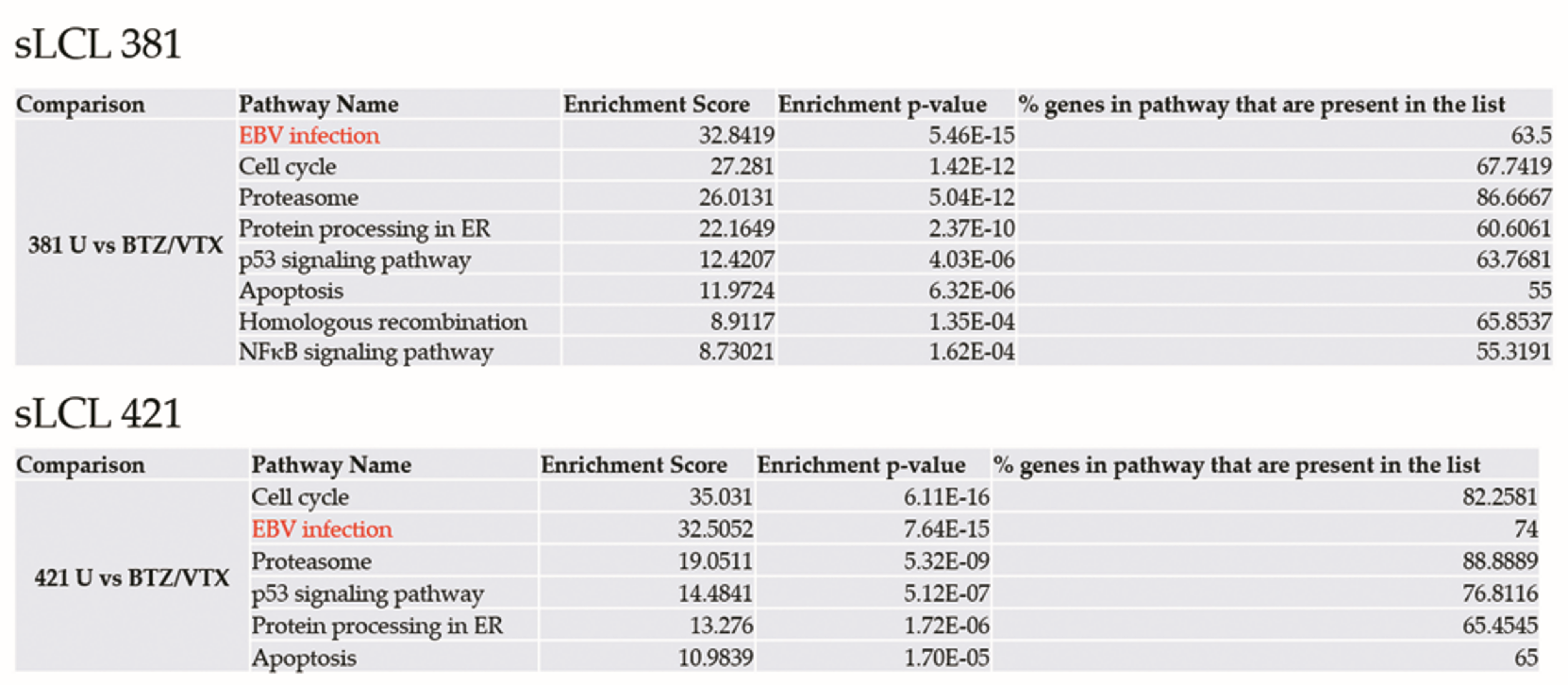

Supplement: S9 Fig — sLCL 381 and sLCL 421 cells were treated with either DMSO or bortezomib/venetoclax for 24 hours. Total RNA was extracted by using TRIzol (Invitrogen, USA). Preparation of cDNA libraries, Illumina sequencing (NovaSeq 6000) and raw data analysis was performed by Centre for PanorOmic Sciences (CPOS), Genomics Core, LKS Faculty of Medicine, The University of Hong Kong. Differentially expressed genes with FDR < 0.05 were adjusted for multiple testing comparisons using Bonferroni’s correction. Pathway enrichment was analyzed by Fisher’s Exact test using Partek Genomics Suite 6.6. (TIF) [file ppat.1012250.s009.tif]

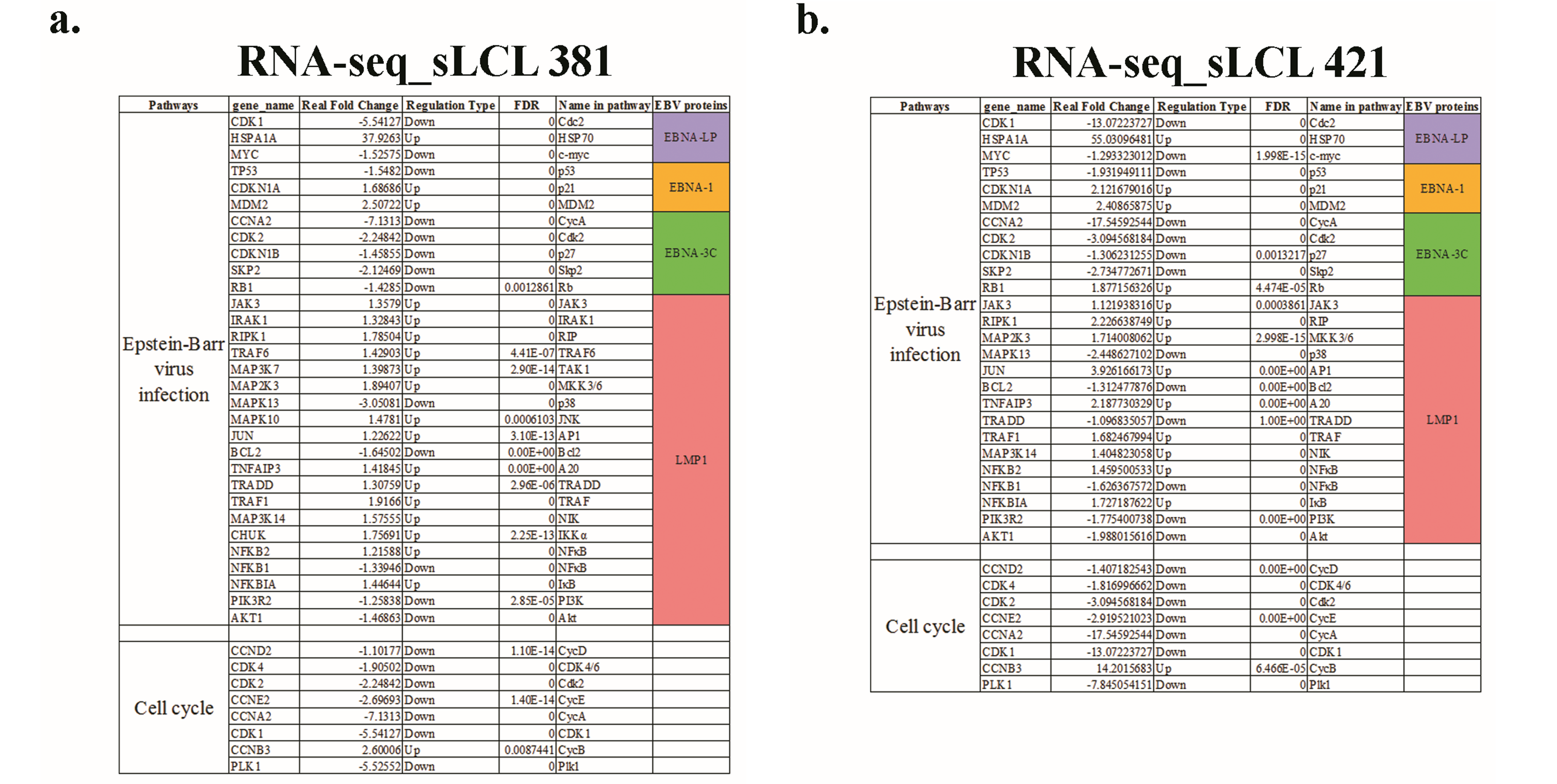

Supplement: S10 Fig — (a) sLCL 381 and (b) sLCL 421 cells were treated with either DMSO or bortezomib/venetoclax for 24 hours. Total RNA was extracted by using TRIzol (Invitrogen, USA). Preparation of cDNA libraries, Illumina sequencing (NovaSeq 6000) and raw data analysis was performed by Centre for PanorOmic Sciences (CPOS), Genomics Core, LKS Faculty of Medicine, The University of Hong Kong. Differentially expressed genes with FDR < 0.05 were adjusted for multiple testing comparisons using Bonferroni’s correction. Pathway enrichment was analyzed by Fisher’s Exact test using Partek Genomics Suite 6.6. The adjusted differentially expressed genes related to EBV proteins and cell cycle were shown. (TIF) [file ppat.1012250.s010.tif]

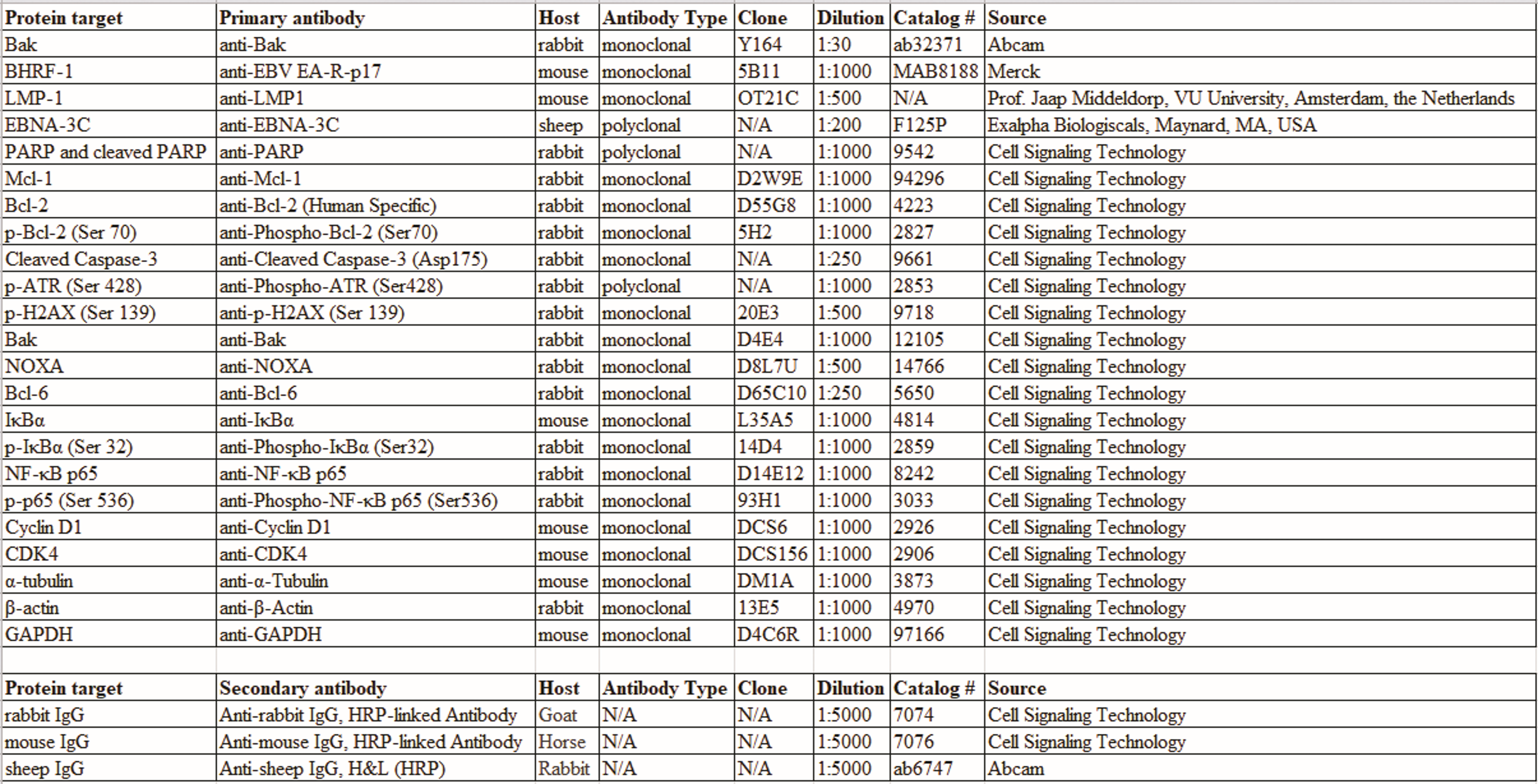

Supplement: S11 Fig — (TIF) [file ppat.1012250.s011.tif]
