# Supplementary material for: Combination of bortezomib and venetoclax targets the pro-survival function of LMP-1 and EBNA-3C of Epstein-Barr virus in spontaneous lymphoblastoid cell lines
Source: PLoS Pathog. 2024 Sep 26;20(9):e1012250. doi: 10.1371/journal.ppat.1012250 (PMC11481030; doi:10.1371/journal.ppat.1012250)
Supplement: Attachment — Submitted filename: Revision1_Point_to_Point_Response_Final.docx [file ppat.1012250.s017.docx]

# Point-to-point response for “Combination of bortezomib and venetoclax targets the pro-survival function of LMP-1 and EBNA-3C of Epstein-Barr virus in spontaneous lymphoblastoid cell lines” by Kam Pui Tam et. al.

**Part I - Summary**

Please use this section to discuss strengths/weaknesses of study, novelty/significance, general execution and scholarship.

***Reviewer #1****: Alan Chiang and colleagues report that a combination of bortezomib and venotoclax can eliminate spontaneous LCLs (sLCLs) established from the blood of patients with PTLD and HLH, both in vitro and in vivo. The rationale behind this combination was to simultaneously induce pro-apoptotic signals mediated by Noxa-1 using bortezomib and to block anti-apoptotic signals such as Mcl-1 and Bcl2 with venotoclax. Furthermore, Bortezomib inhibited proteasome-mediated degradation of I-kappa-B-alpha, thereby maintaining NF-kappa-B inactive and sensitizing cells to apoptotic stimuli.*

*As a result, treated cells showed reduced entry in the cell cycle, increased intrinsic apoptosis and ROS production. Knockdowns of Noxia, BHRF1, EBNA3C and LMP1 showed the importance of these cellular or viral proteins in regulating the LCLs apoptotic status. The authors then used a mouse model to assess the efficacy of the treatment. The combination therapy reduced tumor volume more than 10 times relative to mock controls, and sometimes led to complete regression. There was no evidence of major immediate toxicity associated with the drug treatment.*

*This is a well-written and concise paper that offers new therapeutic perspectives. There is certainly an unmet medical need for these aggressive tumor types and new treatment options using FDA-approved drugs are more than welcome. The authors have shown the efficacy of the combination therapy and offered mechanistic insights to explain the observed effects. Furthermore, the used knockdowns to link these effects with the expression of viral proteins.*

***Reviewer #2****: Strengths- the authors test a drug combination in vitro and in an animal model which suggests that the combination may be of clinical use. They study the effects of the drug combination in a number of in vitro assays to determine the mechanism of action.*

*Weaknesses- some of the findings seem overstated and some controls are missing (see major comments)*

***Reviewer #3****: In their manuscript, Tam and colleagues investigated a potential combination therapy for treating Epstein-Barr virus (EBV) associated post-transplant lymphoproliferative disorder (PTLD) in organ transplant patients. The authors demonstrated a novel treatment strategy involving a combination of bortezomib and venetoclax to target key EBV oncoproteins. They showed promising results in killing patient-derived spontaneous lymphoblastoid cell lines. While the results are compelling and generally support the authors' conclusion, there are a few points that need further experimental support.*

*Specifically:*

*1) The authors should address drug synergy using a more quantitative analysis method, such as SynergyFinder.*

*2) The authors mentioned that the combinatorial treatment induces apoptosis by DNA damage triggered by ROS production. However, they did not provide evidence of increasing levels of DNA damage or ROS production to support their claims, as well as the model proposed in Figure 7. They should also confirm DNA damage in tumor sections from mice treated as in Figure 6.*

**Part II – Major Issues: Key Experiments Required for Acceptance**

Please use this section to detail the key new experiments or modifications of existing experiments that should be absolutely required to validate study conclusions.

Generally, there should be no more than 3 such required experiments or major modifications for a "Major Revision" recommendation. If more than 3 experiments are necessary to validate the study conclusions, then you are encouraged to recommend "Reject".

***Reviewer #1****: 1) What is the evidence that sLCL381 is derived from a PTLD? Under immunosuppression, sLCLs, non-necessarily related to a tumor, are easily established. Thus, the phenotype of the tested sLCLs needs to be better characterized. Does for example this cell line consist of IgM-producing plasmablasts (see doi: 10.1126/scitranslmed.adh8846. Epub 2024 Apr 10.)? Does it carry genetic abnormalities? (See doi: 10.1002/gcc.20287). Are there phenotypic similarities with the clinical tumor they are suspected to derive from? A similar question arises for sLCL421. Does it derive from a histologically proven B cell lymphoma that developed in a patient with HLH? Or is it a non-neoplastic sLCL that grew out in vitro in the context of immune dysregulation and HLH?*

**Author’s response:** Thank you for important comments. We agree with reviewer 1 that the spontaneous LCLs (sLCLs) are derived from EBV transformed B cells rather than the tumor cells of PTLD or EBV-HLH. We state the original clinical diagnosis of T cell PTLD and EBV-HLH at lines 402-406. The sLCLs are generated from bone marrow samples of the patients rather than tumor samples. We state the above at lines 406-408. The flow FISH experiments provide the phenotype of the sLCLs in supplementary figure S1. The sLCLs have high expression of CD19 and are not plasmablasts. Both sLCLs have minimal chromosomal changes by karyotyping; the results are stated at lines 410-412.

***Reviewer #1****: 2) While it is important to test the effect of venotoclax and bortezomib on sLCLs as the authors did in the paper, I would like to see the effect of this drug combination on a set of LCLs established in vitro. Here it would be sufficient to test the optimal concentrations already identified.*

**Author’s response:** The combination of 10nM bortezomib and 5μM venetoclax are identified as the optimum concentration because it is the minimum concentration of each drug to achieve approximately 50% cytotoxicity in both sLCL381 and sLCL421. This is stated at lines 153-156. We test the combination of 10nM bortezomib and 5uM venetoclax on 4 new sLCLs derived from PTLD and EBV-HLH patients. The sLCL information and treatment conditions are stated at lines 402-406 and lines 423-427, respectively. The results show that the combination treatment of 10nM bortezomib and 5uM venetoclax yields similar level of viability (around 50%) in the 4 sLCLs as that of sLCL 381 and sLCL 421. These results are stated at lines 158-160 and demonstrated in supplementary figure S6.

***Reviewer #1****: 3) Similarly, it would be important to delineate the effect of venotoclax and bortezomib on the establishment of LCLs. Transformation assays using primary B cells and laboratory strains in the presence or absence of the drug combination would be suitable.*

**Author’s response:** There will be confounding factors since the transformation of donor’s B cells by B95-8 into LCL may be around 50% in the laboratory. Our main goal is to test the effect of this drug combination in the killing of sLCLs derived from PTLD and EBV-HLH patients.

***Reviewer #2****: 1. The study would benefit from testing of the combination in vitro on EBV-negative B cells. These should be B cells purified from human PBMCs (the vast majority of B cells in the blood are EBV-negative so a seropositive donor would be fine) and an EBV-negative human B cell line(s) (e.g. BJAB, DG75, etc.). This is important to know if the drugs are toxic in the absence of EBV infection.*

**Author’s response:** We test the drug combination on pairs of EBV-negative and EBV-positive BL2 and BL31 (Burkitt) and determine the combination index for synergistic effect. We explain the cell line information and treatment conditions at lines 414-417 and lines 423-427, respectively. We demonstrate that the drug combination elicits stronger synergistic effect on EBV-positive than EBV-negative lines consistently. The results are now described in lines 223-231 and are also shown in supplementary figure S7. The data of shLMP1 and shEBNA3C sLCLs further demonstrate the EBV specificity of this combination treatment as shown in figure 2. In vivo data of stable body weight of vehicle treated mice over a 30-day duration support minimal toxicity of this drug combination as shown in the figure 6d.

***Reviewer #2****: 2. The authors use LCLs from pediatric patients with PTLD. Was permission given by an ethics committee and consent/assent obtain to use these human cells from patients for research? I do not see this in the methods.*

**Author’s response:** We add the human and animal ethics approval numbers at lines 397-400 and at lines 542-544, respectively.

***Reviewer #3****: 1) The authors should address drug synergy using a more quantitative analysis method, such as SynergyFinder.*

**Author’s response:** We use SynergyFinder as suggested by the reviewer for quantitative assessment of the drug synergy. The synergy score of sLCL381 is 16.455 +- 1.23 and that of sLCL421 is 10.289 +- 1.91, supporting that the drug combination has synergistic action. The results are stated at lines 150-153 and are also shown in supplementary figure S8. The synergistic action of bortezomib and venetoclax results in significant tumor growth suppression compared to that of either drug alone in the mice as shown in figure 6. A description of SynergyFinder is added to lines 469-472.

***Reviewer #3****: 2) The authors mentioned that the combinatorial treatment induces apoptosis by DNA damage triggered by ROS production. However, they did not provide evidence of increasing levels of DNA damage or ROS production to support their claims, as well as the model proposed in Figure 7. They should also confirm DNA damage in tumor sections from mice treated as in Figure 6.*

**Author’s response:** The data of TUNEL assay in figure 3 show that the fold change of DNA fragmentation in sLCLs treated with combination treatment is significantly higher than that of single drug treatment. The data of western blot analysis in figures 4b and c show that the expression level of phosphorylated H2AX, a marker for DNA damage, is significantly higher in combinatorial treatment than single drug treatment. In addition, we show that DDR, apoptosis, and cell cycle arrest are diminished when the sLCLs are treated with N-acetylcysteine, a ROS scavenger in figures1c and d. As suggested by the reviewer, we perform immunostaining of tumor sections collected on day 30 from mice using anti-pH2AX antibody. The methods are outlined at lines 553-560. The staining data are added to figure 6 as panel e and f, and the results are stated at lines 295-300. The number of positively stained cells per high power field of combination treatment is significantly higher than vehicle and single treatment. Since there is also significant difference in tumor regression in combination than single treatment (figures 6a and 6b), the quantitation of pH2AX positive cells seems to correlate.

**Part III – Minor Issues: Editorial and Data Presentation Modifications**

Please use this section for editorial suggestions as well as relatively minor modifications of existing data that would enhance clarity.

***Reviewer #2****: 1. The title of the paper and the last sentence of the abstract are misleading. The authors have not tested the drugs in PTLD, only in EBV-transformed B cells from patients with EBV LPD.*

**Author’s response**: We agree with the reviewer. PTLD is changed to spontaneous lymphoblastoid cell lines in the TITLE and the ABSTRACT at line 53.

***Reviewer #2****: 2. line 54. It is simplistic to say rituximab is effective for treatment of PTLD. It works in some, but certainly not all cases (as the authors state later in the paper).*

**Author’s response**: We change from “Rituximab, a chimeric anti-CD20 monoclonal antibody, is effective as single agent or combined with chemotherapy for treatment of PTLD” to “Rituximab, a chimeric anti-CD20 monoclonal antibody, can be an effective agent alone or in combination with chemotherapy in a proportion of the cases of PTLD.” at lines 85-87.

***Reviewer #2****: 3. Line 173 the authors need to indicate what CC3 is.*

**Author’s response**: Stated cleaved caspase 3 (CC3) at line 187.

***Reviewer #2****: 4. Fig 1D. lines 167-168. The authors state that NAC diminished the activity of bort/venetoclax to reduce CDK4 and cyclin D1. This does not seem to be the case in Fig 1D for sLCL421. The bands for the cell cycle proteins do not increase with NAC.*

**Author’s response**: Cyclin D1 and CDK4 show increase after addition of NAC to combination treatment in sLCL381 and to a lesser extent in sLCL421.

***Reviewer #2****: 5. Fig. 2e, Fig.2g. lines 196-198. Knockdown of NOXA does not look like it has much effect on resistance to bortezomib compared to the parental cells (last and first panels in Fig 2e and 2g.*

**Author’s response**: The table below illustrates the actual readings of the viability of the sLCLs treated with increasing concentrations of bortezomib corresponding to the MTT plots of figures 2e and 2g. The viability of shNOXA sLCLs is higher than that of parental sLCLs (particularly at higher concentrations of bortezomib) supporting that shNOXA sLCLs are more resistant to bortezomib.

| ***Bortezomib (nM)*** | ***sLCL381 parental (% viability)*** | ***sLCL381 shNOXA (% viability)*** |
| --- | --- | --- |
| 0 | 100 | 100 |
| 1 | 100.3361 | 100.6035 |
| 2 | 95.43441 | 94.53423 |
| 4 | 93.89231 | 98.34479 |
| 8 | 88.47153 | 89.22307 |
| 16 | 70.49325 | 74.40249 |
| 32 | 47.6594 | 54.94283 |
| 64 | 43.12086 | 54.34159 |

| ***Bortezomib (nM)*** | ***sLCL421 parental (% viability)*** | ***sLCL421 shNOXA (% viability)*** |
| --- | --- | --- |
| 0 | 100 | 100 |
| 1 | 109.2412 | 107.587 |
| 2 | 108.612 | 107.1058 |
| 4 | 105.9556 | 107.5559 |
| 8 | 95.3073 | 96.97176 |
| 16 | 49.52176 | 66.73969 |
| 32 | 28.6775 | 39.10935 |
| 64 | 22.98609 | 28.14535 |

***Reviewer #2****: 6. Line 202 Is a combination index of 0.4869 with shNOXA really different than that of 0.4489 with that of shLUC. Since the shLUC and parental differ by 0.3 this difference seems like it could just be variation in the assay.*

**Author’s response**: We agree that the difference in the CI indices is small. However, the difference of CI between shNOXA and shLuc may still represent a genuine difference because bort/venetoclax-induced DDR, apoptosis and cell cycle arrest are diminished in shNOXA cells as shown in figures 3, 4 and 5. For the experiments, the same shRNA backbone is introduced into both sLCL381 and sLCL421 and the experiments are performed in triplicates.

***Reviewer #2****: 7. Fig 4A, lines 23-224. There is very little difference NF-KB p65 phosphorylation with bort/venetoclax in the either of the shLMP-1 cells. To say there is synergistic reduction is not supported by figure 4A. Similarly lines 225-226 say there is less Bcl-2 and p-Bcl-2(ser) in shLMP1 and shE3C LCLs, but I see little or no difference between bort/venetoclax and any of the other conditions for shE3C sLCL381 cells and for p-Bcl-(Ser) in sLCL381 cells (Fig 4B). I also see no assay for transactivation (line 223). Finally, line 228 refers to Fig 4a and synergy; this figure does not show evidence of synergy.*

**Author’s response**:

Thank you for the reviewer’s detailed comments. We would like to address the points below.

We agree there is NO difference of p-p65 amongst untreated, single or combination treatment of shLMP1 cells (figure 4a). The knockdown of LMP1 in the sLCLs diminishes the effect of NF-kB suppression upon the combination treatment, providing the evidence of targeting of LMP1-induced NF-κB activation by bort/venetoclax.

‘In addition, a marked decrease of Bcl2 and p-Bcl2 in shE3C and shLMP1 cells when compared to the level of Bcl2 in parental, shLuc or other knockdown cells (figures 4b and 4c) indicates that EBNA3C or LMP1 drives Bcl2 expression in sLCLs. This finding is in line with that of previous studies (references 22-24, 32)’. The above sentence is added at lines 248-251 to provide clearer explanation of the data, and the original sentence ‘In addition, less Bcl-2 and p-Bcl-2 (Ser70) was detected……proteasomal degradation of Bcl-6 and IκBα in the sLCLs’ is deleted.

There is no assay for transactivation. We delete the word “transactivation” at line 246.

We change figure 4a to figures 2e-h at line 255 for synergy.

***Reviewer #2****: 8. Figure 4C. line 230-231. While the authors say there is stronger CC3 and c-PARP with bort/venetoclax in parental and shLUC than the shBHRF1, shNOXA, shLMP1 and shE3C cells, figure 4C shows strong CC3, but weaker c-PARP.*

**Author’s response**: We agree and correct cleaved PARP to cleaved PARP/total PARP at line 257.

***Reviewer #2****: 9. Discussion lines 284-285. The authors show no studies of synergy for DNA damage response or inhibition of DNA repair.*

**Author’s response**:

TUNEL assay showed statistically higher DNA fragmentation in combination treatment of sLCLs than in single treatment (figures 3a and 3b). These data support synergistic action of bortezomib and venetoclax in inducing DNA damage. The sentence is revised to accurately say this is ‘DNA damage’ at line 316.

We delete ‘inhibition of DNA repair’ at line 316.

***Reviewer #2****: 10. Discussion lines 310-322. Are the levels of bortezomib cited in these papers similar to the levels of bortezomib used in the current study; if not these studies may not be relevant to the discussion.*

**Author’s response**:

The concentration of bortezomib used in the in-vivo experiment is same as that employed in the referenced paper 38. The concentration of venetoclax is slightly higher than that employed in the referenced paper 39. We consider these two papers are relevant and will retain them in the DISCUSSION at lines 339-353.
